# Genome Landscapes and Bacteriophage Codon Usage

**DOI:** 10.1371/journal.pcbi.1000001

**Published:** 2008-02-29

**Authors:** Julius B. Lucks, David R. Nelson, Grzegorz R. Kudla, Joshua B. Plotkin

**Affiliations:** 1 FAS Center for Systems Biology, Harvard University, Cambridge, Massachusetts, United States of America; 2 Lyman Laboratory of Physics, Harvard University, Cambridge, Massachusetts, United States of America; 3 Department of Biology, University of Pennsylvania, Philadelphia, Pennsylvania, United States of America; Massachusetts Institute of Technology and Harvard University, United States of America

## Abstract

Across all kingdoms of biological life, protein-coding genes exhibit unequal usage of synonymous codons. Although alternative theories abound, translational selection has been accepted as an important mechanism that shapes the patterns of codon usage in prokaryotes and simple eukaryotes. Here we analyze patterns of codon usage across 74 diverse bacteriophages that infect *E. coli*, *P. aeruginosa*, and *L. lactis* as their primary host. We use the concept of a “genome landscape,” which helps reveal non-trivial, long-range patterns in codon usage across a genome. We develop a series of randomization tests that allow us to interrogate the significance of one aspect of codon usage, such as GC content, while controlling for another aspect, such as adaptation to host-preferred codons. We find that 33 phage genomes exhibit highly non-random patterns in their GC3-content, use of host-preferred codons, or both. We show that the head and tail proteins of these phages exhibit significant bias towards host-preferred codons, relative to the non-structural phage proteins. Our results support the hypothesis of translational selection on viral genes for host-preferred codons, over a broad range of bacteriophages.

## Author Summary

Any protein can be encoded by multiple, synonymous spellings. But organisms typically prefer one spelling over another—a phenomenon known as codon bias. Codon bias is generally understood to result from selection for synonymous spellings that increase the rate and accuracy of protein translation. In this work, we have examined the complete genomes of all sequenced viruses that infect the bacteria *E. coli*, *P. aeruginosa*, and *L. lactis*, and have found that many of these viral genomes also exhibit codon bias. Moreover, the degree of codon bias varies across the viral genome, as visualized using a technique called a “genome landscape.” By comparing the observed genomes to randomly drawn genomes, we demonstrate that the regions of high codon bias in these viral genomes often coincide with regions encoding structural proteins. Thus, the proteins that a virus needs to produce in high copy number utilize the same encoding as its host organism does for highly expressed proteins. Our results extend the translational theory of codon bias to the viral kingdom: parts of the viral genome are selected to obey the preferences of its host.

## Introduction

The genomes of most organisms exhibit significant codon bias—that is, the unequal usage of synonymous codons. There are longstanding and contradictory theories to account for such biases. Variation in codon usage between taxa, particularly within mammals, is sometimes attributed to neutral processes—such as mutational biases during DNA replication, repair, and gene conversion [1]–[4].

There are also theories for codon bias driven by selection. Some researchers have discussed codon bias as the result of selection for regulatory function mediated by ribosome pausing [5], or selection against pre-termination codons [6],[7]. However, the dominant selective theory of codon bias in organisms ranging from *E. coli* to *Drosophila* posits that preferred codons correlate with the relative abundances of isoaccepting tRNAs, thereby increasing translational efficiency [8]–[13] and accuracy [14]. This theory helps to explain why codon bias is often more extreme in highly expressed genes [15], or at highly conserved sites within a gene [14]. Translational selection may also explain variation in codon usage between genes selectively expressed in different tissues [16],[17]. However, recent work suggests that synonymous variation, particularly with respect to GC content, affects transcriptional processes as well [18].

The codon usage of viruses has also received considerable attention [19],[20], particularly in the case of bacteriophages [21]–[26]. Most work along these lines has focused on individual phages, or on the patterns of genomic codon usage across a handful of phages of the same host.

Here, we provide a systematic analysis of intragenomic variation in bacteriophage codon usage, using 74 fully sequenced viruses that infect a diverse range of bacterial hosts. Motivated by energy landscapes associated with DNA unzipping [27],[28], we develop a novel methodological tool, called a genome landscape, for studying the long-range properties of codon usage across a phage genome. We introduce a series of randomization tests that isolate different features of codon usage from each other, and from the amino acid sequence of encoded proteins. Thirty-three of the phages in our analysis are shown to exhibit non-random variation in synonymous GC content, as well as non-random variation in codons adapted for host translation, or both. Additionally, we demonstrate that phage genes encoding structural proteins are significantly more adapted to host-preferred codons compared to non-structural genes. We discuss our results in the context of translational selection and lateral gene transfer amongst phages.

## Results

### Genome Landscapes

We start by introducing the concept of a genome landscape, which provides a simple means for visualizing long-range correlations of sequence properties across a genome [29]. A genome landscape is simply a cumulative sum of a specified quantitative property of codons. The calculation of the cumulative sum is straightforward, and it consists of scanning over the genome sequence one codon at a time, gathering the property of each codon, and summing it with the properties of previous codons in the genome sequence. Similar cumulative sums are used in solid-state physics for, e.g., the calculation of energy levels [30]. In the case of the GC3 landscape, we have
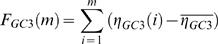
(1)where η*_GC_*_3_(*m*) equals one or zero, depending upon whether the *m^th^* codon ends in a G/C or A/T, respectively. Note that we subtract the genome-wide average GC3 content, 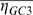, so that *F_GC_*_3_(0) = *F_GC_*_3_(*N*) = 0, where *N* is the length of the genome. In other words, we convert the genome codon sequence into a binary string of 1's and 0's according to whether each codon is of type GC3 or AT3, and we cumulatively sum this sequence to compute *F_GC_*_3_(*m*).

The interpretation of a GC3 landscape is straightforward. Regions of the genome whose landscape exhibits an uphill slope contain higher than average GC3 content, whereas regions of downhill slope contain lower than average GC3 content. The genome landscape provides an efficient visualization of long-range correlations in sequence properties across a genome, similar to the techniques introduced by Karlin [31].

**Figure 1:**
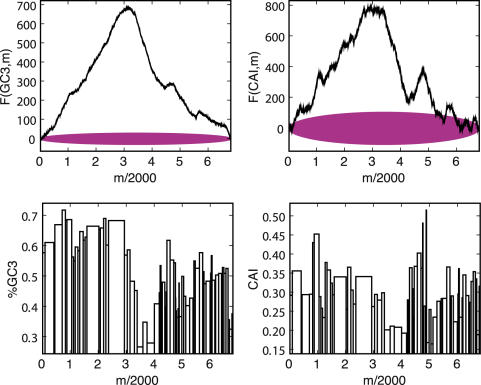
GC3 and CAI landscapes for lambda phage. Landscapes of GC3. (left) and CAI (right) measures of codon usage in Lambda phage. Only coding sequences are considered, which when concatenated together are 40,773 bp long (see Table 2). The GC3 landscape is the mean-centered cumulative sum of the GC3 content (GC3 = 1, AT3 = 0) of codons. The CAI landscape is the mean-centered cumulative sum of the log *w*-value for each codon. For each landscape, a region exhibiting an uphill slope corresponds to higher than average GC3 or CAI. The horizontal purple band represents the expected amount of variation in a random walk of GC3 or AT3 choices, given by Equation 2. Both landscapes exhibit features far outside of the purple bands, indicating that the patterns of codon usage are highly non-random. Gene boundaries are represented by the bars in the histograms below each landscape. The height of the bars in the histogram indicate the GC3 and CAI values for each gene.

Traditional visualizations of GC3 content involve moving window averages of %GC3 over the genome [32]. In order to compare these techniques with the landscape approach, we focus on the *E. coli* phage lambda as an illustrative example. Figure 1A shows the lambda phage GC3 landscape above its associated “GC3 histogram”. The histogram shows the GC3 content of each gene, and the width of each histogram bar reflects the length of the corresponding gene. Thus, the gene-by-gene histograms mimic a sliding window average view of nucleotide content across the genome, but focus on the contributions of individual genes to these sequence properties. Figure 1A reveals a striking pattern of lambda phage codon usage: the genome is apparently divided into two halves that contain significantly different GC3 contents [33],[34]. The large region of uphill slope on the left half of the GC3 landscape reflects the fact that the majority of the genes in this region contain an excess of codons that end in G or C. This trend is also reflected in the GC3 histogram bars, which are higher than average in the left half of the genome (Figure 1).

It is clear that genome landscapes contain the same information as gene-by-gene histograms. However, as has been noted before [29], genome landscapes also represent a powerful visualization tool that emphasizes genome-wide trends in sequence properties. As we demonstrate below, gene-by-gene histograms offer a mechanism by which to quantify these trends, while the landscapes offer striking views of these trends that can aid in their interpretation. In addition, GC-landscapes are directly useful for modeling physical properties of DNA unzipping [28].

Genome landscapes also provide a natural means of evaluating whether or not features of codon usage are due to random chance. Under a null model in which the η(*i*)'s above are chosen as independent random variables with *var*(η(*i*)) = 〈η(*i*)^2^〉−〈η(*i*)^2^〉 = Δ, one can show (see Methods) that the standard deviation of *F*(*GC*3,*m*) is
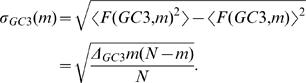
(2)This quantity is shown as a purple band in Figure 1. For η(*i*)'s chosen to be 0 or 1 at random, Δ*_GC_*_3_ = 1/4 and the maximum width 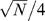 is obtained at *m* = *N*/2. Since the scale of variation across the lambda phage GC3 landscape is much greater than its expectation under the null, we can conclude that the distribution of G/C versus A/T ending codons is highly non-random in the lambda phage genome.

**Figure 2:**
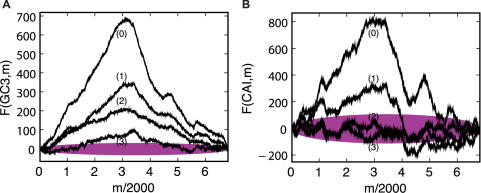
Snapshots of simulated synonymous mutation in the lambda phage genome. (A) Shows GC3 and (B) shows CAI landscapes. In between successive snapshots (labeled by integers), *N* synonymous mutations are introduced into the genome and the resulting landscape is shown, where *N* is the number of codons in the lambda phage genome (see the Genome Landscapes section). These snapshots show that the simulated genome landscapes approach the random null model, indicated by the purple band (see Figure 1). The final CAI landscape (3) lies almost completely within the purple band. Using the lambda phage mutation rate of 7.7×10^−8^ mutations/bp/replication [57], we can estimate that approximately 10^7^ genome replications would be required to relax within the purple bars.

We can also gain intuition about the degree of non-randomness in the GC3 landscape by considering what would happen if the lambda phage genome were to accumulate random synonymous mutations. Figure 2A shows snapshots of the lambda GC3 landscape as we simulate synonymous mutations to the genome. Between each snapshot, *N* synonymous mutations were introduced by picking a codon at random along the genome, and then choosing a new synonymous codon at random according to the global lambda phage codon distribution. By preserving the global codon distribution in each synonymous variation of the genome, this procedure inherently controls for any mutational bias or other source of global codon usage bias that may be present in the phage genome nucleotide content. The same is true for all randomization tests discussed in this paper. As more mutations are introduced, the GC3 landscape of the synonymously mutated lambda genome approaches the purple band, indicating that the GC3 pattern in the real lambda phage genome is highly non-random.

The procedure of producing a genome landscape can be applied to other properties of codon usage. In addition to GC3, we will study patterns in the Codon Adaptation Index (CAI). CAI measures the similarity of a gene's codon usage to the ‘preferred’ codons of an organism [35]—in this case, the host bacterium of the phage under study. Every bacterium has a preferred set of codons defined as the codons, one for each amino acid, that occur most frequently in genes that are translated at high abundance. These genes are often taken to be the ribosomal proteins and translational elongation factors [35] (see Methods).

In order to calculate CAI, the preferred codons are each assigned a weight *w* = 1. The remaining codons are assigned weights according to their frequency in the highly-translated genes, relative to the frequency of the *w* = 1 codon. The CAI of a gene is defined as the geometric mean of the *w*-values for its codons
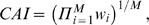
(3)where *w_i_* is the *w*-value of the *i^th^* codon, and *M* is the length of the gene. This quantity can be re-written as
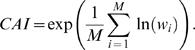
(4)The latter formulation is more useful for calculating genome landscapes, because the argument of the exponential function is now a sum of the logs of the *w*-values. Therefore, we define the CAI landscape as
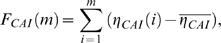
(5)where η*_CAI_*(*m*) = ln(*w_m_*).

The CAI landscape for lambda phage is shown in Figure 1B, along with the CAI histogram of lambda phage. For the CAI histograms, the height of each bar represents the CAI value of that gene (Equation 3). As in the case with the GC3 landscape, we find that the lambda phage CAI landscape corresponds closely to the CAI histogram, but it offers a more striking global view of the long-range CAI structure in the lambda phage genome. One contiguous half of the lambda phage genome exhibits elevated CAI, whereas the other half exhibits depressed CAI. The observed CAI landscape lies far outside the purple band in Figure 1, calculated according to Equation 2, indicating that the pattern of CAI across the lambda phage genome is non-random. However, the purple band is wider for the CAI landscape than for the GC3 landscape, because the variance in the ln(*w_i_*)'s, Δ*_CAI_*, is greater than Δ*_GC_*_3_.

The GC3 and CAI landscapes for lambda phage are highly correlated with each other (Figure 1). In particular they both have large uphill regions on the left-hand side of the genome, indicating a region containing codons with elevated GC3-content and CAI values, compared to the genome average. It is possible that the observed correlation between the GC3 and CAI landscapes could be caused by the conflation between high CAI and GC3 in the preferred *E. coli* codons, as we discuss below.

We note that the genes in the region of elevated CAI primarily encode the highly translated structural proteins that form the capsid and tail of the lambda phage virions. This pattern suggests the hypothesis that, because of the need to produce structural genes in high copy number during the viral life cycle, structural genes preferentially use codons that match the host's preferred set of codons. We will explore this translational-selection hypothesis in greater detail below.

### The Effect of Amino Acid Content on Genome Landscapes

The previous section illustrated that the codon usage across the lambda phage genome is highly non-random with respect to both GC3 and CAI. In this section we quantify this statement, and we focus on aspects of lambda's codon usage patterns that are *independent* of the amino acid sequences of the encoded proteins.

Since we are interested in studying the patterns of *synonymous* codon usage, it is important that we control for the amino acid sequence of encoded proteins. Phages utilize a diverse spectrum of proteins, ranging from those that form the protective capsid for nascent progeny, to those encoding for the tail and tail fibers, to those that regulate the switch between lytic or lysogenic infection pathways. As with other organisms, phage proteins have been selected at the amino acid level for function and folding. Some portion of a phage's codon usage is surely influenced by selection for amino acid content.

We can construct a simple randomization test to interrogate the potential influence of the amino acid sequence on the GC3 and CAI landscapes of lambda phage. In this test, we generate random genomes that have the exact same amino acid sequence as lambda phage, but shuffled codons, such that the genome-wide, or global, codon distribution is preserved in each random genome (see Methods). As summarized in Table 1, we refer to this test as the ‘aqua’ randomization test. For each of the randomized genomes, we calculate GC3 and CAI landscape. Similar to a recent randomization method [36], we then compare the observed landscape of the actual genome to the distribution of landscapes generated from the randomized genomes.

**Table 1:** Randomization test descriptions.

Test Name	Genome Properties Constrained	Genome Properties Varied	Figure
Aqua	Amino acid sequence, global codon distribution	Synonymous codons	3
Orange	Amino acid and BCAI sequences	GC3	5
Green	Amino acid and GC3 sequences	BCAI	5

The three randomization tests used in the paper are color-coded according to what genome properties are constrained in the random trials.

**Table 2:** Phage properties.

Name	Host	Accession	Lifestyle	Number of Genes	Length	Coding Length	Percent GC3	Orange p-value	Green p-value
T5	*E. coli*	NC_005859	NT	161	121750	96051	31.6	1.38×10^−31^	1.71×10^−19^
RB69	*E. coli*	NC_004928	NT	273	167560	156147	29	1.25×10^−21^	5.21×10−^01^
phiEL	*P. aeruginosa*	NC_007623	NT	201	211215	194850	57.8	7.38×10^−20^	2.17×10^−09^
RB49	*E. coli*	NC_005066	NT	273	164018	152592	36.9	2.01×10^−18^	2.48×10^−01^
F116	*P. aeruginosa*	NC_006552	T	70	65195	60240	76.3	1.31×10^−10^	6.31×10^−16^
CTX	*P. aeruginosa*	NC_003278	T	47	35580	31971	81.2	1.44×10^−09^	6.82×10^−32^
phiKMV	*P. aeruginosa*	NC_005045	NT	49	42519	38310	79.9	3.25×10^−09^	9.54×10^−03^
T4	*E. coli*	NC_000866	NT	269	168903	153660	24.3	4.59×10^−09^	8.62×10^−01^
lambda	*E. coli*	NC_001416	T	69	48502	40773	53.5	6.25×10^−09^	5.10×10^−68^
D3	*P. aeruginosa*	NC_002484	T	94	56425	49095	68.3	1.57×10^−08^	3.85×10^−07^
P2	*E. coli*	NC_001895	T	42	33593	30411	54.7	5.60×10^−08^	2.54×10^−61^
P1	*E. coli*	NC_005856	T	108	94800	80103	48.2	9.37×10^−08^	3.51×10^−11^
D3112	*P. aeruginosa*	NC_005178	T	55	37611	34908	80.4	3.05×10^−07^	4.35×10^−05^
WPhi	*E. coli*	NC_005056	T	43	32684	29601	56.4	8.39×10^−07^	7.80×10^−55^
K1F	*E. coli*	NC_007456	NT	43	39704	34629	53.4	1.75×10^−05^	8.03×10^−02^
T3	*E. coli*	NC_003298	NT	47	38208	29694	54.3	3.50×10^−05^	3.07×10^−04^
PaP3	*P. aeruginosa*	NC_004466	T	71	45503	41115	58.1	5.09×10^−05^	1.64×10^−19^
phiV10	*E. coli*	NC_007804	T	55	39104	36111	48.8	1.25×10^−04^	9.38×10^−11^
P27	*E. coli*	NC_003356	T	58	42575	37707	50.5	2.24×10^−04^	2.23×10^−20^
933W	*E. coli*	NC_000924	T	78	61670	52956	50	4.29×10^−04^	8.88×10^−09^
B3	*P. aeruginosa*	NC_006548	T	56	38439	36138	77.3	4.40×10^−04^	3.33×10^−05^
HK97	*E. coli*	NC_002167	T	59	39732	34191	52.1	7.61×10^−04^	1.19×10^−20^
VT2-Sa	*E. coli*	NC_000902	T	83	60942	52647	51.3	1.31×10^−03^	7.40×10^−07^
PRD1	*E. coli*	NC_001421	NT	21	14925	11988	47.6	2.99×10^−03^	5.97×10^−02^
JK06	*E. coli*	NC_007291	U	71	46072	32841	43	3.84×10^−03^	1.63×10^−03^
T1	*E. coli*	NC_005833	NT	77	48836	44010	47.7	7.45×10^−03^	3.64×10^−01^
Pf1	*P. aeruginosa*	NC_001331	U	12	7349	6282	75.7	9.66×10^−03^	6.67×10^−01^
HK022	*E. coli*	NC_002166	T	57	40751	33885	52.7	1.25×10^−02^	4.36×10^−18^
4268	*L. lactis*	NC_004746	NT	49	36596	33759	24.7	1.59×10^−02^	3.20×10^−01^
BP-4795	*E. coli*	NC_004813	T	48	57930	22356	48.1	1.66×10^−02^	3.29×10^−10^
186	*E. coli*	NC_001317	T	43	30624	27747	58.7	4.02×10^−02^	1.79×10^−22^
I2-2	*E. coli*	NC_001332	U	8	6744	5166	35	6.91×10^−02^	1.01×10^−01^
phiKZ	*P. aeruginosa*	NC_004629	NT	306	280334	243384	26.8	1.32×10^−01^	1.79×10^−14^
bIL312	*L. lactis*	NC_002671	T	27	15179	11292	28.1	1.49×10^−01^	8.85×10^−04^
HK620	*E. coli*	NC_002730	T	58	38297	33717	45.9	1.61×10^−01^	1.41×10^−05^
Mu	*E. coli*	NC_000929	T	54	36717	33900	54.1	1.68×10^−01^	4.49×10^−10^
P4	*E. coli*	NC_001609	T	14	11624	9765	52.4	1.71×10^−01^	4.17×10^−18^
N15	*E. coli*	NC_001901	T	59	46375	41472	54.9	2.17×10^−01^	1.38×10^−09^
Stx2 I	*E. coli*	NC_003525	T	97	61765	34932	48.4	3.04×10^−01^	4.23×10^−04^
bIL286	*L. lactis*	NC_002667	T	61	41834	38694	24.8	3.68×10^−01^	1.17×10^−01^
Tuc2009	*L. lactis*	NC_002703	T	56	38347	35178	28	4.08×10^−01^	1.81×10^−02^
Stx2 II	*E. coli*	NC_004914	T	99	62706	34755	50.1	5.85×10^−01^	9.94×10^−03^
BK5-T	*L. lactis*	NC_002796	T	52	40003	33267	24	5.91×10^−01^	6.68×10^−01^
Stx1	*E. coli*	NC_004913	T	93	59866	33444	49.5	6.75×10^−01^	2.97×10^−03^
LC3	*L. lactis*	NC_005822	T	51	32172	29607	24.6	7.31×10^−01^	4.90×10^−01^
ul36	*L. lactis*	NC_004066	NT	58	36798	32400	27.7	8.64×10^−01^	4.66×10^−02^
Pf3	*P. aeruginosa*	NC_001418	U	9	5833	5487	35.9	8.70×10^−01^	1.64×10^−06^
bIL285	*L. lactis*	NC_002666	T	62	35538	32646	26.7	9.20×10^−01^	9.93×10^−01^
r1t	*L. lactis*	NC_004302	T	50	33350	30315	25.4	9.53×10^−01^	6.03×10^−01^
bIL170	*L. lactis*	NC_001909	T	63	31754	27663	27.1	9.91×10^−01^	8.71×10^−01^

Properties are listed for all phages included in Figure 8, in the same order based on the orange *p*-value. Lifestyle annotations are T (temperate), NT (non-temperate), U (unknown). The coding length refers to the length of all coding sequences concatenated together (see Methods).

**Figure 3:**
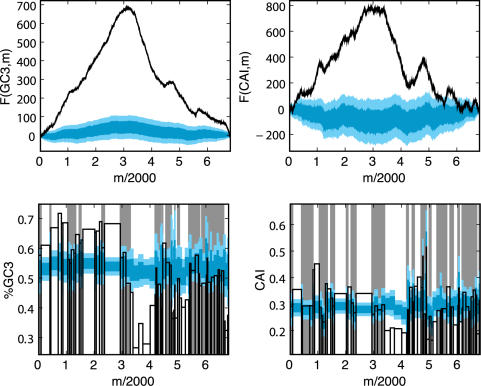
Observed and randomized landscapes for lambda phage. The figure shows the observed GC3 (left) and CAI (right) landscapes, plotted in black, along with the mean±1, and ±2 standard deviations of randomized trials, shown in aqua (bold line, dark and light regions, respectively). The aqua randomization test shown here draws random synonymous codons that preserve the exact amino acid sequence, according to probabilities that preserve the global codon usage distribution of the lambda genome. For the most part, the observed landscapes lie significantly outside the distribution of randomized landscapes–implying that the amino acid content of genes is not responsible for the observed pattern of the CAI landscape. In the lower panel, however, genes whose GC3 (left) or CAI (right) values fall between the 0.025 and 0.975 quantile of the random trials are shadowed in grey; the GC3/CAI values of such genes are not significantly different from random, given their amino acid sequence.

Figure 3 shows the results of this comparison, with the observed landscapes plotted as black lines, and the mean±one and two standard deviations of random trials shown in dark and light aqua, respectively. As the figures show, the observed landscapes lie in the far extremes of the randomized distributions – indicating that the amino acid sequence of the lambda phage genome does not determine the extraordinary features of the observed landscapes.

It is also instructive to query the influence of amino acid content on codon usage in each gene individually. The histogram view of these randomization tests allows us to ask this question precisely. Because the amino acid sequence is preserved exactly across the genome, each histogram bar in Figure 3 can be considered as its own randomization test, one for each gene. The position of the horizontal black bar reflects the actual codon usage of each gene, and it can be compared to the distribution of random trials in order to compute a quantile for each gene:
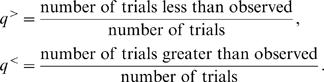
(6)Note that we have defined two quantiles, *q* ^>^ and *q* ^<^, that describe the proportion of random trials strictly less or strictly greater than the observed data. These two quantities sum to a values less than one (and equal to one if there are no ties). A value of *q* ^>^>0.5 signifies that the observed statistic (e.g. GC3 or CAI) is *greater* than most of the random trials.

Associated with each of these quantiles is a p-value quantifying whether the observed gene sequence has significantly different codon usage than the random trials: *p* ^<^ = 1−*q* ^<^ and *p* ^>^ = 1−*q* ^>^. If either one of these *p*-values is low, it signifies that the GC3 (or CAI) content of the gene is significantly different than the genomic average, controlling for the amino acid sequence of the gene. *p* ^<^ tests for significantly depressed GC3 (or CAI) in a gene; and *p* ^>^ tests for significantly elevated GC3 (or CAI) in a gene. We will use these *p*-values, which arise from the ‘aqua’ randomization test, in two ways.

Since we are interested in studying the effects of synonymous codon usage alone, we first wish to filter out any genes whose codon usage does not significantly deviate from random, given the amino acid sequence. Therefore, in the subsequent gene-by-gene analyses reported in this paper, we retain only those genes whose quantiles fall in the extreme 5% of random trials. That is, we only keep those genes for which 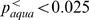 or 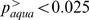. These genes are said to ‘pass’ the aqua test, and they are unshaded in Figure 3.

We also use the gene-by-gene *p*-values to quantify the degree to which codon usage is independent of amino acid sequence across the genome as a whole. To do so, we combine all the gene-by-gene *p*-values into an aggregate *p*-value for the entire genome, *p_aqua_*, using the method of Fisher [37]. We calculate the combined *p*-value by summing the logs of twice the minimum of each gene-specific p-value
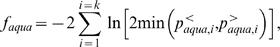
(7)where 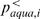 represents the aqua *p* ^<^-value for gene *i*, and *k* is the number of genes in the genome. It is well known that *f_aqua_* is chi-squared distributed with 2*k* degrees of freedom [37]. Thus, the combined *p*-value for the entire genome, 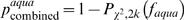, where 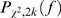 is the cumulative chi-squared distribution with 2*k* degrees of freedom. In the case of lambda phage, we find 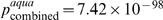 for GC3 and 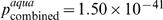 for CAI. Thus, we conclude that the neither the GC3 nor the CAI patterns across the lambda phage genome are determined by the genome's amino acid sequence.

In the following sections we will use the aqua test (see Table 1) and its associated gene-by-gene and combined p-values as a control to verify that features of codon usage are not driven by the amino acid sequence.

### Disentangling CAI from GC3

**Figure 4:**
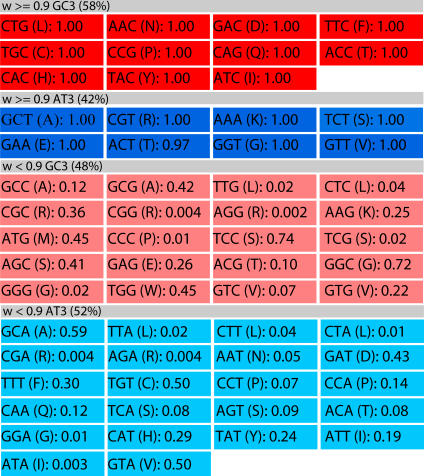
*E. coli* codon usage master table. The table of 61 codons along with their associated *w*-values is shown for *E. coli*. The *w*-value of each codon reflects its frequency in highly transcribed *E. coli* genes (see main text). The Table 1 is divided into four regions: codons with high CAI (w≥0.9) ending in G or C (dark red); codons with high CAI ending in A or T (dark blue); codons with low CAI (*w*<0.9) ending in G or C (light red); codons with low CAI ending in A or T (light blue). As the table shows, there is a slight bias for GC3 in the high-CAI codons (58%), and slight bias away from GC3 in the low-CAI codons (48%).

Depending upon the preferred codons of the host species, the effect of selection for high CAI in a viral gene is not necessarily independent from the effect of selection for other features of viral codon usage, such as high GC3. For example, codons with high CAI values associated with a given host may be biased towards high GC3 values as well (see Figure 4). It is important, therefore, to disentangle the effects of selection for CAI versus selection for GC3, in order to determine which one of these forces is responsible for the non-random patterns of codon usage observed in the lambda genome.

The weights used to compute CAI for *E. coli* are shown in Figure 4. The 61 codons are placed into one of four groups according to whether they are GC3 or not (red or blue, respectively), and whether they have high CAI or not (dark or light, respectively). High CAI is determined by an arbitrary cutoff of *w*≥0.9. As this table demonstrates, the set of preferred codons in E. coli is slightly biased towards GC-ending codons (58%).

The GC bias of preferred codons, although slight, could conflate the results of selection for CAI versus GC3 in phages that infect *E. coli*, such as lambda. We therefore introduce another randomization test that allows us to disentangle patterns of CAI content from patterns of GC3 content. Similar to the aqua randomization test described above, we draw random phage genomes such that the amino acid sequence is conserved, but we add the additional constraint of conserving the exact GC3 sequence as well (see Methods). For example, at a site containing a GC3 codon for leucine, in our random trials we only allow those leucine codons terminating in G or C. By comparing the observed landscapes of the genome with the distribution of randomly drawn landscapes, we can isolate the features of codon usage driven by CAI, independent of GC3 and amino acid content. We refer to this randomization procedure at the ‘orange’ randomization test (Table 1).

Conversely, we also wish to assess the strength of patterns in GC3 content, independent of CAI and amino acid content. The appropriate randomization procedure in this case requires that we constrain the amino acid sequence and the sequence of codon CAI values while allowing GC3 to vary. However, because CAI values are not binary, CAI cannot be constrained exactly while still allowing for enough variability to produce a meaningful randomization test. Thus, we introduce a binary version of the CAI measure, called BCAI, that is qualitatively the same as and, for our purposes, interchangeable with CAI.

The BCAI *w*-value for a codon is defined to be 0.7 if the codon is high CAI, and 0.3 if the codon has low CAI. High CAI is defined by the threshold of *w*≥0.9 (see Figure 4). The threshold value *w*≥0.9 is arbitrary, and our results are robust to changing this threshold (see Figures S1 and S2). Our use of the term ‘binary’ here refers to the binary classification scheme and not the particular values of BCAI. The actual values assigned for BCAI are arbitrary, for the most part, and have no effect on our results. Nevertheless, we cannot assign low BCAI a value of zero, because this value would be problematic when included in the geometric averaging procedure, or when computing the logarithm of w-values for BCAI landscapes.

**Figure 5:**
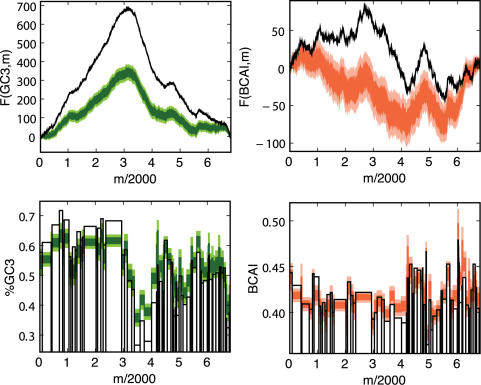
Observed and randomized landscapes for lambda phage. Observed landscapes are shown along with randomized landscapes associated with the green and orange tests. The green randomization procedure tests the significance of the GC3 landscape controlling for the observed CAI (actually, BCAI) variation across the genome. The orange randomization procedure tests the significance of the BCAI landscape, controlling for the observed GC3 variation across the genome. Both tests preserve the amino-acid sequence exactly. Both observed landscapes lie outside the distribution of random trials, indicating there is non-random GC3 content controlling for CAI, and non-random CAI content controlling for GC3.

BCAI provides a useful surrogate for CAI because its values are binary, thereby allowing us to constrain a gene's amino acid sequence and BCAI sequence *exactly*, while varying GC3 content in random trials. The BCAI landscapes and histograms are calculated in the same way as CAI landscapes and histograms, except using BCAI *w*-values. As expected, the BCAI landscape of a genome is qualitatively similar to its CAI landscape (compare Figures 5B and 3B), and the two landscapes are highly correlated (e.g. *r* = 0.72 for lambda phage). Thus BCAI is interchangeable with CAI for the purposes of our randomization tests.

Figure 5 shows the results of the two randomization tests outlined above: the ‘green’ test that compares the observed GC3 landscape to a distribution of random trials constraining the amino acid sequence and the BCAI sequence; and the ‘orange’ test that compares the observed BCAI landscape to a distribution of random trials constraining the amino acid sequence and the GC3 sequence. Our convention for naming these two tests is summarized in Table 1.

As seen in Figure 5A, the observed GC3 landscape lies significantly outside of the random trials that preserve amino acid sequence and BCAI sequence. Combining the gene-by-gene p-values for this test, we find 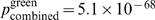 – indicating that the lambda phage genome as a whole has non-random GC3 variation independent of amino acid and CAI (actually, BCAI) sequence. Conversely, Figure 5B shows that the BCAI landscape contains non-random features when controlling for both GC3 and amino acid sequence (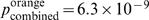). In other words, the lambda phage genome exhibits highly non-random patterns of both GC3 and CAI codon variation, independent of one another and independent of the amino acid sequence.

### Non-Random Patterns of CAI and GC3 in Bacteriophages

In the sections above we have demonstrated and quantified highly non-random patterns of GC3 and CAI codon usage variation across the lambda phage genome. We have also demonstrated that these trends are independent of one another. In this section, we will extend our analysis to a large range of diverse phages.

**Figure 6:**
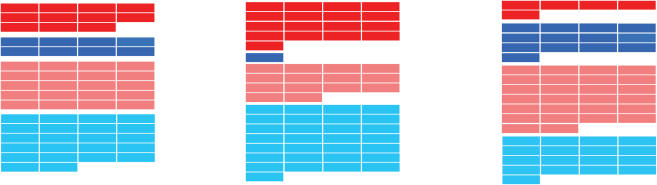
Schematics of preferred codon usage tables for *E. coli*, *P. aeruginosa*, and *L. lactis* following the conventions of Figure 4. Unlike *E. coli*, *P. aeruginosa* strongly favors GC3 in high-CAI codons (94%), and *L. lactis* strongly favors AT3 in high-CAI codons (72%).

In this section we consider all sequenced phages that infect *E. coli*, *Pseudomonas aeruginosa* or *Lactococcus lactis* as their primary host. The latter two hosts were chosen because of they contain unusually extreme GC3 content: 88 %GC3 for *P. aeurginosa* and 25 %GC3 for *L. lactis*, genome-wide. The extreme GC3 content of these hosts give rise to opposing relationships between high CAI and GC3 – as indicated schematically in Figure 6. In particular, *P. aeruginosa* strongly favors GC3 in high-CAI codons (94%), and *L. lactis* strongly favors AT3 in high-CAI codons (72%). Thus, these three hosts span a large spectrum of relationships between CAI and GC3. Since our randomization tests constrain amino acid and BCAI exactly (the ‘green’ test), and amino acids and GC3 exactly (the ‘orange’ test), we can control for any possible conflation between GC3 and CAI trends. Thus, the randomization tests are equally applicable to all of the phage genomes, regardless of their host.

We performed the aqua, green, and orange randomization tests on the 45 phages of *E. coli*, 12 phages of *P. aeruginosa*, and 17 phages of *L. lactis* whose genomes have been sequenced (see Methods). In the first step of our analysis, we removed any phages which failed either the aqua GC3 or aqua CAI tests, because the codon usage of such genomes are influenced by their amino acid sequence. A phage was said to pass these two control tests if its Fisher combined p-values for both aqua GC3 and aqua CAI were significant. The significance criterion for each test is *p*_combined_<5%/74, which incorporates a Bonferroni correction for multiple tests. With this cutoff, 50 of the initial 74 phages passed the aqua control tests.

**Figure 7:**
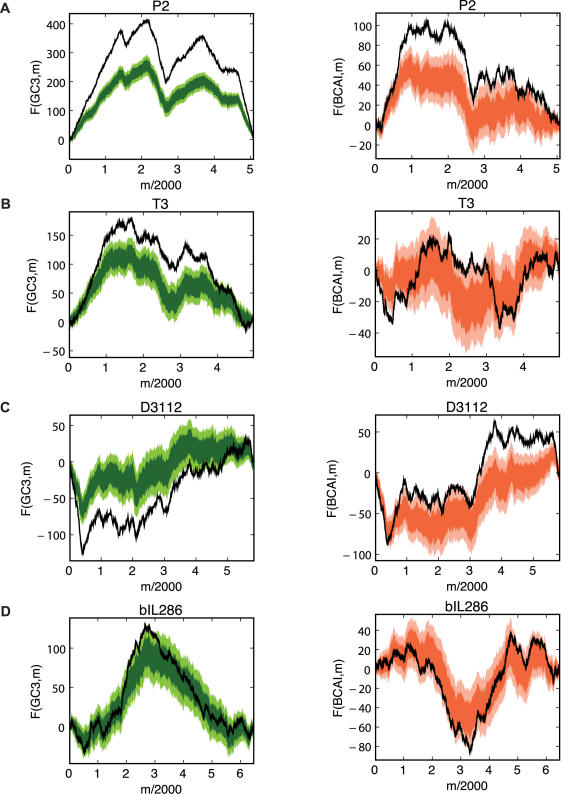
Green (left) and orange (right) randomization tests for several phages. Bacteriophages P2 (A) and T3 (B) both infect *E. coli*. Phage D3112 (C) infects *P. aeruginosa*. Phage bIL286 (D) infects *L. lactis*. T3 is the only non-temperate phage of this group. See Table 2 for combined Fisher p-values for these tests. In the case of bIL286, note the lack of evidence for codon bias evident in the green and orange tests for bIL286, as confirmed by the insignificant *p*-values in Table 2. In this case, we cannot rule out the possibility that the observed pattern in GC3 is determined completely by the amino acid and CAI sequence (green), or that the observed pattern in CAI is determined by the amino acid and GC3 sequence (orange).

Figure 7 shows results of these tests for several example genomes. P2, a temperate phage, and T3, a non-temperate phage both infect *E. coli* and both pass the control tests and exhibit significant ‘orange’ and ‘green’ results, as does D3112, a temperate phage that infects *P. aeruginosa*. However, not all phages that pass the control test exhibit significant ‘orange’ and ‘green’ results – as evidenced by bIL286, a temperate phage infecting *L. lactis*.

**Figure 8:**
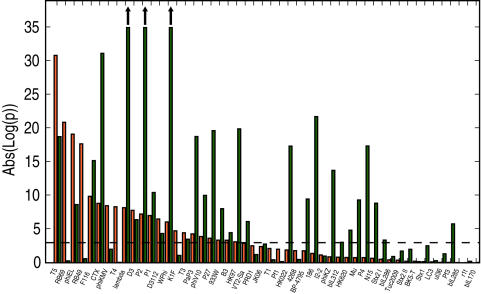
Combined Fisher p-values for the green and orange randomization tests across 50 phage genomes. Phage names are listed on the x-axis, and are sorted by their orange *p*-value. A total of 29 genomes exhibit non-random GC3 content controlling for CAI (green test); and a total of 22 genome exhibit non-random CAI content controlling for GC3 (orange test). 17 genomes pass both of these tests. The dashed horizontal line indicates the threshold for significance after Bonfernni correction (i.e. 5%/50). Upwards arrows indicate p-values that lie beyond the limits of the y-axis. See Table 2 for phage properties, including the *p*-values for these tests. Twenty four phage genomes that failed the aqua GC3 or CAI control tests are not included in this figure.

Figure 8 plots the distribution of combined Fisher p-values of the orange and green tests, for the 50 phages that pass the control tests. The majority of these p-values are highly significant. Using a Bonferoni-corrected threshold of 5%/50, a total of 22 genomes show significance in the orange test, 29 in the green test, and 17 in both orange and green. These results indicate that non-random patterns in codon usage are not unique to lambda phage. Indeed, over a range of bacterial hosts and a range of phage viruses, there is apparent pressure for non-random patterns of both GC3 content and CAI content, independent of one another and independent of the amino acid sequence.

### Translational Selection on Phage Structural Proteins

In this section, we investigate a natural hypothesis concerning the patterns of non-random CAI usage we have observed in phage genomes – namely, that these patterns may be driven by selection for translational accuracy and efficiency, which is stronger in more highly expressed proteins [9],[21].

Among all phage proteins, the structural proteins are the most highly expressed [38]. The structural proteins form the protective capsid that encloses the viral genome, as well as the tail, which is often used for transmission of the phage genome to the inside of the host [39]. These proteins must be produced in high copy number – many tens of copies of each type of structural protein needed to form each of hundreds of viral progeny [38]. For each gene in a phage genome, we assigned a structural annotation of 1 if the gene was known to encode a structural protein and 0 otherwise (see Methods).

According to the standard hypothesis of translational selection, the structural genes of phages should exhibit elevated CAI levels compared to other phage genes, since they are translated (by the host) in high copy numbers. To test this hypothesis, we performed regressions between the structural annotation of phage genes and their aqua CAI and orange BCAI p-values. In other words, we compared the structural properties of genes against their CAI content, controlling for amino acid sequence, and against their BCAI content, controlling for both amino acid sequence and GC3 sequence.

**Figure 9:**
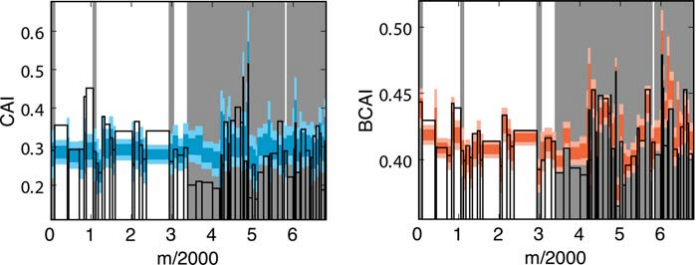
The relationship between codon usage and protein function in lambda phage. The figure shows the aqua (CAI, as in Figure 3) and orange (BCAI, as in Figure 5) randomization tests overlaid with information about protein function: genes classified as structural are shown with a white background and all other genes with a grey background. The histograms indicate a clear relationship between the structural classification of a gene and its significance under the aqua and orange tests: structural genes typically have elevated quantiles in the aqua test, whereas other genes typically have depressed quantiles. In other words, structural genes exhibit elevated CAI values when controlling for their amino acid sequence, compared to codon usage in the genome as a whole. Moreover, as the orange histograms indicate, this pattern is not caused by variation in GC3 content: the structural genes exhibit elevated BCAI values after controlling for both their amino acid sequence and their GC3 sequence.

In the case of lambda phage, Figure 9 shows the results of the aqua CAI and orange BCAI randomization tests, with the structural genes highlighted. The plot reveals a striking pattern: the vast majority of the structural proteins lie on the left half of the genome, exactly in the region where genes have elevated CAI values. In order to quantify this association we performed ANOVAs. Before regressing structural annotations against codon usage, we first removed the non-informative genes – i.e. genes whose codon usage are influenced by their amino acid content, as indicated by a failure to pass the aqua CAI test.

Table 3 shows the results of the regression between aqua CAI and orange BCAI *p* ^>^-values versus structural annotations in lambda phage. The results are highly significant: structural annotations explain half of the variation in CAI, even when controlling for genes' amino acid sequences (aqua, *r* ^2^ = 56%) as well as GC3 sequences (orange test, *r* ^2^ = 46%). The median *p* ^>^-value among structural genes is close to zero, whereas the median *p* ^>^-value among non-structural genes is close to one – indicating that structural genes exhibit significantly *elevated* CAI values. These highly significant results are consistent with the hypothesis of translational selection on structural proteins.

**Table 3:** Structural annotation verses codon usage.

Structure/Non-Structure/Test		Lambda	All Phage Genes
Number structural		7	279
Number non-structural		18	1022
Aqua CAI Randomization Test	median p^>^ structural	1.3×10^−4^	8.0×10^−3^
	median p^>^ non-structural	1	1
	ANOVA significance	p = 4.5×10^−5^	p = 4.7×10^−12^
Orange BCAI Randomization Test	median p^>^ structural	2.8×10^−2^	2.0×10^−1^
	median p^>^ non-structural	0.98	0.73
	ANOVA significance	p = 1.8×10^−4^	p = 1.6×10^−15^

The table shows the median *p* ^>^ values among structural and non-structural genes, under the aqua and orange randomization tests. Small *p* ^>^ values indicate significantly elevated CAI, controlling for the amino acid sequence (aqua test) and the GC3 sequence (orange test). We also report the significance of non-parametric ANOVAs that compare median *p* ^>^-values between the structural and non-structural genes. Analyses are limited to those genes that pass the aqua test, as described in the main text; similar results are found without this restriction.

**Table 4:** Comparison between codon usage and refined structural annotations.

Number/Test		All Phage Genes
Number Head		145
Number Tail		134
Number non-structural (NS)		1022
CAI Randomization Test	median p^>^ head	2.0×10^−3^
	median p^>^ tail	2.0×10^−2^
	median p^>^ NS	1
	ANOVA Head vs NS	p = 6.4×10^−19^
	ANOVA Tail vs NS	p = 1.8×10^−1^
	ANOVA Head vs Tail	p = 2.1×10^−8^
Orange BCAI Randomization Test	median p^>^ head	7.0×10^−2^
	median p^>^ tail	4.3×10^−1^
	median p^>^ NS	0.73
	ANOVA Head vs NS	p = 4.2×10^−21^
	ANOVA Tail vs NS	p = 1.7×10^−2^
	ANOVA Head vs Tail	p = 6.0×10^−8^

As in Table 3, we compare the median aqua and orange *p* ^>^ values among head genes, tail genes, and non-structural genes. We report the significance of pairwise non-parametric ANOVAs comparing head to non-structural, tail to non-structural, and head to tail genes. These analyses are limited to genes that pass the aqua test; similar results are found without this restriction.

In order to examine the relationship between structural annotation and CAI across all 74 phages in our study, we performed the same ANOVA on the 1,309 informative genes (i.e. genes that pass the aqua CAI randomization test). Once again, Table 3 shows a highly significant relationship between structural annotation and CAI values, controlling for amino acid content and GC3. Thus, the tendency toward elevated CAI values in structural genes holds across all the phages in this study, despite the fact that they infect a diverse range of hosts with a wide variety of GC contents.

Similar to reports for other organisms [40], we find a relationship between gene length and codon adaptation. In our case, however, longer viral genes are associated with more significant *p* ^>^-values in the aqua and orange tests. However, the strength of this relationship is weak, and controlling for gene length does not affect our results on elevated CAI in structural proteins (ANOVA *p*-values analogous to Table 3 are less than 10^−9^ after controlling for gene length).

## Discussion

In this paper, we have developed genome landscapes as a tool for visualizing and analyzing long-range patterns of codon usage across a genome. In combination with a series of randomization tests, we have applied this tool to study synonymous codon usage in 74 fully sequenced phages that infect a diverse range of bacterial hosts. Genome landscapes provide a convenient means to identify long-range trends that are not apparent through conventional, gene-by-gene or moving-window analyses. Using a statistical test that compares codon usage to random trials, controlling for the amino acid sequence, we found that we found that many of the phages studied exhibit non-random variation in codon usage. However, not all of the phages exhibit non-random variation as exemplified by phage bIL286 (Figure 7D).

In light of long-standing [9] and recent [18] literature from other organisms, we have focused on two aspects of phage codon usage: variation in third-position GC/AT content (GC3) and variation in the degree of adaptation to the ‘preferred’ codons of the host (CAI). Almost three-quarters of the phages in our study exhibit non-random intragenomic patterns of codon usage, even when controlling for the amino acid sequence encoded by the genome. Almost half of such genomes also show non-random patterns of CAI when additionally controlling for the GC3 sequence. In other words, there is substantial variation in CAI above and beyond what would be expected by random chance, given the amino acid and GC3 sequences of these genomes.

We have also compared the CAI values of phage genes to their annotations as structural or non-structural proteins. We have conclusively demonstrated that phage genes encoding structural proteins exhibit significantly elevated CAI values compared to the non-structural proteins from the same genome. These results hold even when controlling for the amino acid sequence and GC3 sequence of genes. Our conclusions across a diverse range of phages are consistent with early observations on lambda's codon usage [34], early results for T7 [21], and with the general hypothesis of translational selection, which predicts elevated CAI in genes expressed at high levels [9],[15],[35]. The pattern of elevated CAI in structural proteins is particularly striking the case of lambda phage. It is also worth noting that we find no significant relationship between a phage's life-history (i.e. temperate versus non-temperate) and the degree to which its structural proteins exhibit elevated CAI (see Table 6). This observation likely reflects the fact that at some point every phage, regardless of its life history, must generate certain structural proteins in high abundance – and so it is beneficial to encode such protein using the host's translationally preferred codons.

**Table 5:** The number of tRNA genes in phage genomes.

Phage	Number of tRNAs in genome
T5	25
T4	8
VT2-Sa	3
933W	3
Phi 186	1
D3	4
P27	2
PaP3	4
RB69	2

For each phage genome, the GenBank entry was scanned for the presence of tRNA genes. The number of these genes are listed beside the names of the phages for the ten phage genomes in this study that do encode tRNAs.

**Table 6:** Phage lifestyle versus codon usage.

Phage		Significance
Median 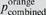	Temperate	1.4×10^−2^
	Non-temperate	2.6×10^−5^
	Un-identified	4×10^−2^
	ANOVA significance	p = 0.1
Median 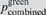	Temperate	5.1×10^−9^
	Non-temperate	7.0×10^−2^
	Un-identified	5×10^−2^
	ANOVA significance	p = 0.009

The table shows the median 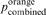 and 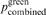 values among phages classified as temperate, non-temperate, or un-identified for all phages included in Figure 8 and Table 2. Small median 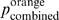 values indicate that these phages have significantly non-random (in either direction) BCAI, controlling for the amino acid sequence and the GC3 sequence, while small median 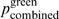 values indicate that these phages have significantly non-random (in either direction) GC3, controlling for the amino acid sequence and the BCAI sequence. We also report the significance of non-parametric ANOVAs that compare these medians between these groups of phages.

Some of the phages examined are known to encode their own tRNA genes. Table 5 lists the number of tRNA genes for the ten phages in this study that encode tRNA genes. We have inspected these examples for signs that structural genes might be preferentially encoded by endogenous tRNAs, or the converse, but have concluded that the data are equivocal. There are too few informative examples to make a strong conclusion in either direction.

Our results on translational selection in phages shed light on the nature of selection on viruses. The standard interpretation of elevated CAI in highly expressed bacterial proteins assumes a fitness cost (per molecule) associated with inefficient or inaccurate translation. We have observed a similar relationship between expression level and CAI across a diverse range of bacteriophages, which presumably do not incur a direct energetic cost from inefficient translation by their hosts. Thus, our results suggest that either there is an adaptive benefit (to the virus) of elevated CAI in phage structural proteins, or that costs incurred by the host bacterium also reduce the fitness of the virus.

In addition to our results on CAI, we have also observed non-random patterns of GC3 variation across the genomes of many phages. These patterns are highly significant even after controlling for potential conflating factors, such as the amino acid sequences and CAI sequences of genes. Unlike our results on CAI, there is no clear mechanistic hypothesis underlying the non-random patterns of GC3 in phages. It is possible that these patterns reflect selection for efficient transcription [18] or for mRNA secondary structure. But in the absence of independent information on such constraints, we cannot assess the merits of these selective hypotheses, nor rule out the possibility of variation in mutational biases across the phage genomes. It is interesting to note that we find these significant non-random patterns of GC3 predominantly in temperate phages (see Table 6).

Our study benefits from the number and breadth of phages we have analyzed. Unlike previous studies, here we analyze phages whose suspected hosts span a diverse range of bacteria, which themselves differ in their genomic GC3 content and preferred codon choice. We have calibrated CAI for each phage according to its primary host, and nevertheless we find consistent relationships between CAI and viral protein function. These results therefore conclusively extend the classical theory of translational selection to the relationship between viruses and their hosts.

The present study also benefits from the development of randomization tests that isolate the patterns of variation in CAI from variation in GC content. Due to intrinsic biases in the GC content of the preferred codons of hosts, previously studies on codon usage in phage have conflated these two types of synonymous variation [23]–[26]. The mechanisms underlying GC3 variation and CAI variation likely differ, and so it is critically important that we have analyzed each of these features controlling for the other one.

There is a large literature on the structure and evolution of phage genomes which is pertinent to our analyses of phage codon usage. The genomes of phages that infect *E. coli*, *L. lactis*, and *Mycobacteria* are known to be highly mosaic in structure [41]–[46]. In other words, these genomes exhibit many similar local features that suggest each genome was assembled from a common pool of bacteriophage genomic regions [47]. Recently, mosaicism was discussed in the lambdoid phages focusing specifically on the *E. coli* phages lambda, HK97 and N15 [38]. We note that both HK97 and N15 have peaked landscape structures like lambda, although not as pronounced, indicating that some degree of mosaicism can be observed in genome landscapes among closely related phages. The postulated mechanism for mosaicism is homologous and non-homologus recombination between co-infecting phages or between a phage and a prophage embedded in the host genome [42],[47],[48]. Some have argued that the latter mechanism occurs more frequently, due to the large number of lysogenized prophages in bacterial genomes [48].

Lateral gene transfers could affect the codon usage patterns of phages, especially if recombination occurs between phages whose preferred hosts differ. In this case, the codon usage patterns of each phage may be expected to reflect the preferred codons of their preferred hosts; a recent recombination may result in regions of dramatically different codon usage from the average phage codon usage. In particular, regions of unusual GC3 content in a phage genome could reflect gene transfers between phages that typically infect hosts of different GC3 content, in analogy with lateral gene transfer amongst bacteria [49]. Morons are genes in phage genomes that are under different transcriptional control than the rest of the phage genes, and are often expressed when the phage is in the lysogenic state [50]. These morons have been observed to have very different nucleotide compositions compared to the rest of the phage genome suggesting that they are the result of such gene transfers [50]. Thus one interpretation for our observations of the 29 phages exhibiting non-random GC3 patterns is that these genomes arose through recent recombination events, and have not subsequently experienced enough time to equilibrate their GC3 content to that of their current host. Given the lack of reliable estimates for time scales between putative phage recombination events, or for codon usage equilibration, this study neither supports nor refutes this interpretation. However, the predominance of significant non-random patterns of GC3 in the genomes of temperate phages (see Table 6) suggest that such recombination may occur more frequently among temperate phage populations.

We have demonstrated that phage genes encoding structural proteins exhibit significantly elevated CAI values compared the non-structural phage genes. These results support the classical translation selection hypothesis, now extended to the relationship between viral and host codon usage. We do not find much variation in codon usage among the structural genes themselves. This observation has two plausible interpretations within the literature of lateral gene transfers: either phages of different preferred hosts rarely co-infect, or there is substantially less recombination among the structural proteins of phages. The latter hypothesis has been independently suggested for the capsid proteins of phages, based on the idea that capsid proteins form a complex with multiple physical interactions whose function would be disrupted by individual gene transfer events [43]. Unlike capsid genes, phage tail genes often exhibit mosaicism, and they can include elements from diverse viruses with variable host ranges [43],[51]. To investigate this phenomenon in the context of codon usage, we refined the structural annotation to separate head from tail genes (see Methods). We performed three separate ANOVAs to compare the CAI usage in these genes: comparing head versus non-structural, tail versus non-structural, and head versus tail (Table 4). These regressions indicate that the head genes are primarily responsible for that pattern of elevated CAI in structural proteins. In addition, we detect a difference in codon usage between head and tail genes. These results have at least two possible explanations: either the head proteins are produced in higher copy number than the tail proteins, or lateral gene transfers between diverse phages occur frequently enough in the tail genes to impair their ability to optimize codon usage to their current host. The first hypothesis is very plausible, in light of evidence on the copy number of head and tail proteins [38]; nevertheless, we cannot rule out the second possibility.

Finally we note that our methodologies could offer a mechanism to analyze the recently growing amount of phage DNA sequences gathered through metagenomic studies [52],[53]. We have shown that, especially for genes encoding structural proteins, there is a strong host-specific signature in the viral genome – namely the enrichment of host-preferred codons. Raw metagenomic data seldom identify the relationship between the viral DNA segments sequenced and the hosts they infect. We may be able to help glean such information using a form of the randomization tests developed here to search over all possible host master tables, identifying potential hosts as those that maximize the statistical significance of the randomization tests.

## Materials and Methods

### Bacteriophage Genomes

Bacteriophage genomes were downloaded from NCBI's GenBank (http://www.ncbi.nlm.nih.gov/Genbank/index.html) release 156 (October, 2006) using Biopython's [54] NCBI interface. We only used reference sequence (refseq) phage genome records with accessions of the form NC_00dddd in order to have the most complete records available. Of the 396 phage refseq's available, we focused on the 74 genomes of phages whose primary host, as listed in the specific_host tag in the Genbank file, were *E. coli*, *P. aeruginosa* or *L. lactis*.

All phage genomes were downloaded from GenBank. Before being used for the rest of this study, every gene within a genome was scanned for overlaps within other genes in the same genome, and all overlapping sequences were removed. A codon was only retained if all three of its nucleotides occurred in a single open reading frame. Thus the final genome sequence used was a concatenation of all non-overlapping coding sequences, omitting any control elements and other non-coding sequences.

### Calculation of CAI Master Tables

The definition of the Codon Adaptation Index requires the construction of a ‘master’ *w*-table for the host organism. Each of the 61 sense codons is assigned a *w*-value based on the codon's frequency among the most highly expressed genes in the host organism. In defining this set of genes, we follow Sharp [35], who specified highly expressed genes for *E. coli*.

In order to calculate the CAI master *w*-tables for *P. aeruginosa* and *L. lactis*, we identified the homologs of the highly expressed *E. coli* genes within the other host genomes, using BLAST [55]. In particular, we used qblast to find homologs to these *E. coli* genes by inputting the gene protein sequences, and blasting (blastp) against the nr database, restricting the database to include proteins of the target organism. In all cases, we used the most significant blast result as the ortholog, provided its e-value was less than 1×10^−10^.

Given the set of highly expressed genes, the CAI master *w*-table was calculated as follows. For each host, the GenBank file (GenBank release 156) was downloaded locally and transformed into a local data structure using Biopython's [54] GenBank parser. The data structure was then scanned for each of the genes in the highly translated gene set, and the collective CDS codon sequences of these genes were concatenated together into one long sequence. Stop codons and codons encoding for amino acids methionine (M), and tryptophan (W) (each encoded by only one codon) were removed from the concatenated sequence. The frequencies of codons encoding all other amino acids were then tabulated, and divided into groups according to which amino acid they encode. The w-values are then calculated, according to the procedure of Sharp [35], as these frequencies, normalized by the maximum frequency within each group. Thus each amino acid has a codon with a *w*-value of 1, representing the most commonly used codon for that amino acid. The *w*-values for the stop codons and codons for methionine and tryptophan were set to the average w-value of the remaining codons.

### Drawing Random Genomes According to Constraints

Our randomization tests require drawing randomized phage genomes that are constrained to have specific properties. In all of the randomization tests discussed, the random sequences were drawn as a sequence of synonymous codons from the global codon distribution at each position, thereby exactly preserving the amino acid sequences of proteins. Furthermore, each test preserves the global codon distribution in each synonymous variation of the genome, and thus inherently controls for any mutational bias or other source of global codon usage bias that may be present in the phage genome nucleotide content. The tests thus isolate the feature that we wish to interrogate which is local patterns in synonymous codon usage.

The three randomization tests used in this work can all be considered variants of a canonical randomization test that preserves both the amino acid sequence and a bit mask sequence exactly, while drawing codons from the global, genome-wide distribution. A bit mask sequence is string of zeros and ones corresponding to all codons in the genome. For example, GC3 is 1 if the third position of a codon is G or C, and 0 otherwise.

Using the GC3 bit mask as an example, the randomization test procedure is initialized by calculating the global codon frequencies that fit into categories specified by the amino acid and the bit-mask value. Each amino acid has associated with it two distributions: one for a bit-mask value of 1 and one for a bit-mask value of 0. For example, alanine (A), is encoded by four codons, GCC (1), GCG (1), GCT (0), GCA (0), where the GC3 bit-mask is shown in parenthesis. Thus to calculate the codon distribution of alanine GC3 codons (*A*_1_), we compute the frequency of GCC and GCG codons across the whole phage genome. Similarly, the distribution of *A*_0_ codons is determined from the frequency of GCT and GCA codons across the genome. In order to produce a random genome, random codons are drawn at each position according to the distribution associated with the position's amino acid and bit-mask value.

Thus the three null tests can be specified by the definition of the bit mask along the sequence, which determines the constraints on the randomize trials. The aqua randomization test constrains the amino acid sequence and nothing else, and so its bit mask consists of all 1's. The orange randomization test preserves the amino acid and the GC3, and so its bit mask is the GC3 sequence mentioned above. The green randomization test preserves the amino acid and BCAI exactly, thus its bit mask is the thresholded BCAI (1 if BCAI = 0.7, 0 otherwise).

In considering the power of the green and orange randomization tests, we must ask how many synonymous families permit one to constrain BCAI and change the last codon position from G/C to A/T. The answer to this question depends upon the CAI master table of the host species. For E. coli (see Figure 4), all nine the 3-, 4-, and 6-fold degenerate codon families permit one to constrain BCAI (at 0.3) while varying G/C to A/T. However, constraining BCAI typically determines GC3 for the 2-fold degenerate families. As a result, roughly 60% of the codons in a phage genome are informative for the green randomization test. Similar results hold for *P. aeriginosa* and *L. lactis*, and for the orange test.

For both of these tests, even if few synonmous families were informative, this feature would serve to weaken the power of statistics, making our conclusions conservative.

### Structural Annotation

All phage genes were annotated as structural or non-structural by inspecting the annotations of high-scoring BLAST hits among viral proteins. This procedure is described in detail below.

Each gene was considered separately within each genome object, although overlaps were removed in the process of creating the genome objects. The amino acid sequence of each gene was blasted against all known viral protein sequences using Biopython's interface [54] to the NCBI blast utility [55]. Specifically, we used the blastp utility specifying the nr database, with entrez query ‘Viruses [ORGN]’. We retained only those BLAST hits with e-values below the cutoff 1×10^−4^. All words in the title of these BLAST hits were collected, using white space as a word-delimiter.

The unique words from the blast hits were then compared against a set of structural keywords: “capsid”, “structural”, “head”, “tail”, “fiber”, “scaffold”, “portal”, “coat”, and “tape”. The words associated with the BLAST hits were scanned for matches to the keywords, where each keyword was treated as a regular expression. As a result, partial matching was counted as a match. For example, a BLAST title containing the word ‘head-tail’ would match both keywords ‘head’ and ‘tail’. If a gene had at least one structural keyword match in its BLAST hit title, it was annotated as structural. Otherwise, it was annotated as non-structural.

We further subdivided the structural annotation into two classes: head and tail genes. Tail genes were identified with the keywords “tail”, “fiber”, and “tape”. These remaining structural genes that did not contain any of these keywords were annotated as head genes. Two false positives for tail identification in the lambda phage genome were manually corrected.

### Null Model: Results for Random Walk Landscapes

In the sections above we have compared the genome landscapes calculated from real genome sequences to a null model in which the sequences are randomly drawn from a defined distribution. In this section, we compute several properties of genome landscapes calculated from these random genomes.

We write the general genome landscape of length *N* as
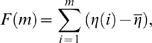
(8)where η(*i*) are independent, and chosen from a random distribution with *var*(η(*i*)) = 〈η(*i*)^2^〉−〈η(*i*)〉^2^ = Δ, and
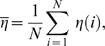
(9)which ensures *F*(0) = *F*(*N*) = 0.

The purple regions in Figure 1 represent the variance in the genome landscapes of this null model at each *m*, 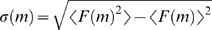. Using the definitions above, we have
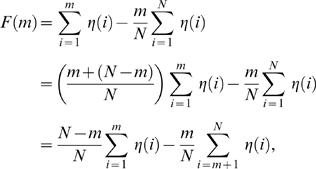
(10)and
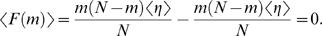
(11)When we use 〈η(*i*)η(*j*)〉 = 〈η^2^〉δ*_i_*_,*j*_+(1−δ*_i_*_,*j*_)〈η〉^2^, with δ*_i_*_,*j*_ = 1 if *i* = *j* and 0 otherwise, we find
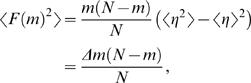
(12)leading to 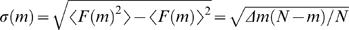. In the case of GC3 landscapes, η(*i*) is either 1 or 0 with equal probability, giving Δ*_GC_*_3_ = 1/4.

We can also calculate the full probability distribution, *P*(*f*;*m*,*N*,Δ) that the genome landscape of length *N* has an intermediate value *F*(*m*) = *f*, at point *m*, by considering an *N*-step random walk that is constrained to start and stop at 0. This probability distribution can be written as a product of two conditional probabilities for a walk that starts at 0 and ends at *f* in *m* steps, and a walk that starts at *f* and ends at 0 in *N*−*m* steps
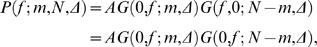
(13)where *A* is a normalization constant, and the last step used the inversion symmetry of the random walks. Thus we seek the form of the conditional probability *G*(0,*f*;*m*,Δ). In the same way as in Equation 13, we decompose this conditional probability into a multiplication of the conditional probabilities for two walks, one that starts at 0 and ends at y in *x* steps, and one that starts at *y* and ends at *f* in *m*−*x* steps, and integrate over all possible intermediate values *y*

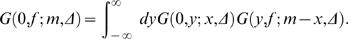
(14)We can continue this decomposition for each intermediate step to give
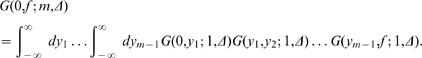
(15)Keeping the order of integration the same, and noting that *G*(*y*_1_,*y*_2_;1,Δ) = *G*(*y*_2_−*y*_1_;1,Δ) for these random walks, we can write *y_i_*_+1_−*y_i_* = *s_i_*_+1_ to give
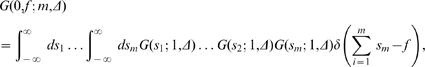
(16)where the delta function is added to force the constraint that the sum of all the intermediate steps must be equal to *f*. All of the intermediate conditional probabilities now represent one step walks, and so are equal to the underlying probability distribution of drawing a step size *s_m_*, *p*(*s_m_*;Δ)
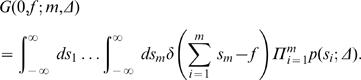
(17)Making use of the integral representation of the delta function [56]

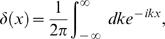
(18)we have
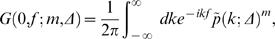
(19)where 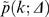 is the Fourier transform of *p*(*s*,Δ)
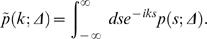
(20)For the purpose of this discussion, we assume *p*(*s*,Δ) has a Gaussian form 
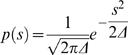
, and note that the results are general. In this case, 
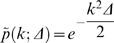
, and we have
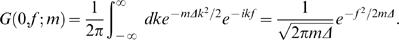
(21)To determine *A*, we enforce the normalization condition
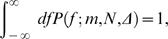
(22)which gives
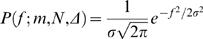
(23)

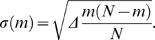
(24)Note that from the full distribution, we can immediately identify 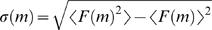, confirming the explicit calculation above.

## Supporting Information

Figure S1. Orange randomization test for the lambda phage genome with a BCAI cutoff of c = 0.5.
As expected, the only qualitative difference between this figure and Figure 5 in the paper is the scale on the y-axis.
(0.46 MB EPS)

Figure S2 Lambda Phage BCAI landscapes for different cutoff values.
Lamba phage BCAI landscapes for different cutoffs, c, where we have assigned codons with w≥c a value of BCAI = 0.7, and w<c a value of BCAI = 0.3. Note that the landscapes are qualitatively the same and only differ in y-scale. As we expect, the smaller c, the more BCAI = 0.7 codons, and thus the large the y-scale of the landscapes.
(0.75 MB EPS)
